# Does Combined Fractionated Stereotactic Radiotherapy and Immunotherapy Change the Outcome of Recurrent High-Grade Gliomas?

**DOI:** 10.7759/cureus.15852

**Published:** 2021-06-23

**Authors:** Sezin Yuce Sari, Burak Yasin Aktas, Neyran Kertmen, Aysenur Elmali, Sadettin Kilickap, Kader Karli Oguz, Melike Mut, Mustafa Erman, Figen Soylemezoglu, Faruk Zorlu, Gozde Yazici

**Affiliations:** 1 Radiation Oncology, Hacettepe University Medical School, Ankara, TUR; 2 Medical Oncology, Hacettepe University Medical School, Ankara, TUR; 3 Radiology, Hacettepe University Medical School, Ankara, TUR; 4 Neurosurgery, Hacettepe University Medical School, Ankara, TUR; 5 Pathology, Hacettepe University Medical School, Ankara, TUR

**Keywords:** fractionated stereotactic radiotherapy, immunotherapy, immunoradiotherapy, re-irradiation, recurrent high-grade glioma

## Abstract

Background

Radiotherapy (RT) with immune checkpoint inhibitors (ICI) has yielded good responses in many cancers. We aimed to report the results of combined fractionated stereotactic radiotherapy (FSRT) and ICI in patients with recurrent high-grade glioma.

Methodology

Patients were treated with FSRT and nivolumab which were continued until progression or toxicity. The Response Assessment in Neuro-oncology and Immunotherapy Response Assessment in Neuro-oncology criteria were used to assess treatment response on magnetic resonance imaging. Treatment-related toxicity was noted in all patients.

Results

A total of eight patients were included. Recurrence was detected after a median of 5.8 months following the first RT, all in the treatment field. FSRT (3 × 8 Gy) was applied with neoadjuvant, concurrent, and adjuvant nivolumab. After a median follow-up of 21.3 months from diagnosis and 12.6 months from recurrence, one patient was alive and seven succumbed to the disease. The median overall survival was 20.9 months after diagnosis and 12.9 months after recurrence. The median progression-free interval was 2.3 months after FSRT. The local control (LC) rate was 62.5% with a median local recurrence-free survival of nine months. Progression in other regions of the brain was observed in four patients with a median progression-free survival of 2.1 months. Acute toxicity was not observed. ICI-related grade 3 late pneumonitis was observed in two patients, and grade 1 late thyroid toxicity in two patients. One patient with pneumonitis also developed osteoporosis and radiation necrosis.

Conclusions

A high LC rate was achieved with concurrent FSRT and ICI with a severe late toxicity rate of 25%. This combination can be an option in recurrent high-grade gliomas.

## Introduction

The treatment of choice for high-grade gliomas is maximal surgical resection followed by external beam radiotherapy (EBRT) with concurrent and adjuvant temozolomide (TMZ) for 6-12 cycles [1,2]. Although adding bevacizumab is contradictory for increased neurocognitive deficits and other severe adverse events in de-novo glioblastoma (GB), some clinicians prefer it in recurrence for its durable radiographic response and increased disease-free survival (DFS) with no proven overall survival (OS) benefit [3,4]. Despite advances in treatment, the recurrence rate is still high in high-grade gliomas. After recurrence, although not always possible, a second surgery is preferred with increased survival rates [5]. Salvage re-irradiation increases OS and progression-free survival (PFS) rates in GB [6-8]. Recently, immunotherapy with immune checkpoint inhibitors (ICI) has been introduced for many tumors with poor survival rates as well as high-grade gliomas.

Combined ICI and radiotherapy (RT) increases survival rates in many tumors. The data on ICI mostly come from melanoma and other solid tumors; however, the exact involvement of checkpoint pathways in central nervous system (CNS) tumor pathogenesis is unknown. RT causes immunogenic cell death and can improve the efficacy of ICI. In murine models of GB, combined ICI and RT increased the survival rate [9]. A prospective study on the combined use of ICI and RT has been recently published [10]. In this study, 5 × 6 Gy fractionated stereotactic radiotherapy (FSRT) was used with pembrolizumab and bevacizumab with fairly increased OS rates compared to the studies without ICI. Although the control rate was high, the details regarding when and where these patients recurred were not provided. Other prospective studies of programmed death-ligand (PD-L) 1 inhibitors with or without RT are ongoing for de novo and recurrent GB.

In light of sparse data, we report our results in patients with recurrent high-grade gliomas treated with combined ICI and RT in our center. The purpose of this study is to report the outcomes and toxicity in these patients. In addition, we aim to clearly define the recurrence pattern when ICI is added to FSRT.

## Materials and methods

Treatment and follow-up schedule

Patients ≥18 years of age with a diagnosis of recurrent high-grade glioma who were treated with FSRT and concurrent ICI were included in this study. All patients had undergone gross total (GTR) or subtotal resection (STR) after physical examination, blood parameters testing, and brain magnetic resonance imaging (MRI), and were diagnosed with high-grade glioma. EBRT was administered with or without concurrent and adjuvant TMZ. All patients were followed up with brain MRI every three months. Progression was diagnosed and distinguished from radiation necrosis based on contrast-enhanced T1-weighted, diffusion-weighted, and perfusion MRI and magnetic resonance spectroscopy. Histological confirmation was not required. Consequently, all patients underwent FSRT with concurrent ICI. Patients with tumors in close proximity to the organs at risk (OAR) were included in this second RT phase. Subsequently, patients were followed up with brain MRI every month during the first three months, and then after every three months.

All patients were informed about the experimental role of the ICI and its potential toxicity, following which they signed an informed consent form prior to ICI and FSRT. This study was approved by Hacettepe University Ethics Committee for Non-Invasive Clinical Research.

Treatment at first diagnosis

After the first surgery, concurrent EBRT+TMZ was planned in all patients. Patients were treated with intensity-modulated radiotherapy (IMRT) or volumetric modulated arc therapy (VMAT) via Elekta Synergy Platform (Elekta AB, Stockholm, Sweden) or Varian Clinac DHX High Performance (Varian Medical Systems Inc., Palo Alto, CA, USA). The gross tumor volume (GTV) was defined as the contrast-enhancing volume on T1 images of postoperative MRI. The clinical target volume (CTV)-1 and CTV-2 were defined as GTV and the hyperintense volume on T2 or fluid-attenuated inversion recovery images plus a 2.5 cm and a 1 cm margin, respectively. Patient number 3 received hypofractionated EBRT with a single CTV (GTV+1 cm). The planning target volume (PTV) was defined as CTV+5 mm. CTV-1 and CTV-2 were prescribed at 40 and 60 Gy, respectively. TMZ was administered at a dose of 75 mg/m^2^ concurrently with EBRT daily including weekends, and adjuvant TMZ was administered at a dose of 150 mg/m^2^ on days one to five every 28 days until progression.

Treatment at progression

Progression was defined as the increase in contrast enhancement or the size of the residual tumor or newly detected contrast-enhanced lesion on follow-up MRI. All patients were evaluated for surgery. All patients underwent FSRT irrespective of surgery status. A thermoplastic mask was used for immobilization in the supine position. Planning computed tomography (CT) was performed with a slice thickness of 1 mm starting from the tip of the cranium to the bottom level of the cervical vertebrae after administration of 100 cc intravenous contrast. The MRI at the time of recurrence was fused to the planning CT. The GTV was the contrast-enhanced recurrent tumor on T1-weighted images of the MRI. The CTV was equal to the GTV with the addition of the tumor bed after the second operation if present. No additional margin was considered for the PTV. OARs were also contoured in each slice. The cumulative equivalent uniform dose in 2-Gy fraction was kept at a maximum of <80 Gy for the optic structures and <90 Gy for the brain stem, with a median <90-100 Gy for the brain [11]. FSRT was administered two days a week via CyberKnife® (Accuray, Sunnyvale, CA, USA). Treatment plans were optimized so that the whole PTV received 100% of the prescription dose. However, the coverage aim was switched to ≥95% of the PTV receiving 95% of the prescribed dose when the PTV was in close proximity to OARs. Six-D skull tracking was used during treatment.

All patients received intravenous 3 mg/kg nivolumab (a PD-1 inhibitor) neoadjuvant to, concurrently with, and adjuvant to FSRT. Nivolumab was administered in over 60 minutes every 14 days, with 28 days constituting one cycle. Patients were continued on nivolumab until progression or toxicity.

The Response Assessment in Neuro-oncology (RANO) criteria were used to assess treatment response [12]. Complete response (CR) was defined as the disappearance of the tumor, partial response (PR) as a >50% reduction, stable disease as <50% reduction or <25% increase, and progressive disease as a >25% increase in tumor size on consecutive MRIs at least one month apart. In addition, based on the Immunotherapy Response Assessment in Neuro-oncology (iRANO) criteria, progressive disease or appearance of new lesions within six months of ICI initiation does not necessarily point to a true progression [13]. Thus, patients continued receiving ICI as long as they tolerated it.

Statistical analysis

All statistical analyses were performed using the Statistical Package for the Social Sciences (SPSS) version 18.0 (SPSS Inc., Chicago, IL, USA). The primary endpoints were PFS and local control (LC). The secondary endpoints were OS, local recurrence-free survival (LRFS), and treatment-related toxicity. LC was defined as no relapse of the tumor following FSRT in the prior FSRT field. We defined OS as the time from diagnosis and from the date of recurrence after FSRT to the last follow-up or death from any cause, PFS as the time from the date of FSRT and from the start of nivolumab to the date of appearance of any recurrence or death from any cause, and LRFS as the time from the date of FSRT to the date of LR or death from any cause. Survival analyses were carried out using the Kaplan-Meier method. The incidence of toxicity was defined as the total number of patients reaching that grade at any time.

## Results

A total of eight patients (six females and two males) were treated with FSRT and ICI between January 2019 and March 2020. The median age was 52 years (range: 44-74 years). Of the total patients, seven had undergone STR and one GTR at first diagnosis. Histopathology revealed anaplastic astrocytoma (AA) in one, gliosarcoma (GS) in one, and GB in six patients based on the 2016 World Health Organization classification [14]. Isocitrate dehydrogenase-1 (IDH-1) was positive in two patients. Moreover, codeletion of 1p19q was present in the patient with AA. In the four patients in whom the O^6^-methylguanine methyltransferase (MGMT) status could be tested, MGMT was methylated in patients 1 and 3 and unmethylated in patients 2 and 8. A retrospective mismatch repair analysis was performed for six patients whose surgery was performed in our hospital. In patients 3, 5, 7, and 8, PMS2, MLH1, MSH6, and MSH2 were diffusely positive. In patient number 2, PMS2 and MSH6 were focally positive but MLH1 and MSH2 were diffusely positive. Lastly, in patient number 6, MSH6 was focally positive while the others were diffusely positive. Six patients could receive the planned adjuvant scheme. Patient number 3 with poor performance and postoperative meningitis received 40 Gy in 2.66 Gy fractions without TMZ. Patient number 6 received 52 Gy EBRT and four weeks of TMZ due to persistent thrombocytopenia. Patient and treatment details are listed in Table 1.

**Table 1:** Patient and treatment characteristics. HP: histopathology; adj: adjuvant; tx: treatment; TTP: time to progression; ICI: immune checkpoint inhibitor; F: female; M: male; GTR: gross total resection; STR: subtotal resection; AA: anaplastic astrocytoma; GB: glioblastoma; GS: gliosarcoma; CCRT: concurrent chemoradiotherapy; TMZ: temozolomide; RT: radiotherapy; B-I: bevacizumab+irinotecan; FSRT: fractionated stereotactic radiotherapy ^a^after the end of external beam radiotherapy ^b^after the end of FSRT

Patient number	Gender/Age (year)	Surgery type/date	HP	Adj tx	TTP 1^st ^(months)^a^	Progression tx	Local response	TTP 2^nd^ (months)^b^
1	F/50	GTR/October 2016	AA	CCRT + adj TMZ (×12)	17	B-I (×7), STR + ICI (×16) + FSRT	Progression	3
2	F/62	STR/March 2018	GB	CCRT + adj TMZ (×7)	11	ICI (×7) + FSRT	Partial response	-
3	M/74	STR/February 2018	GB	RT	7	B-I + ICI (×8) + FSRT	Partial response	-
4	F/64	STR/May 2018	GB	CCRT + adj TMZ (×3)	5	ICI (×6) + FSRT	Partial response	-
5	F/44	STR/September 2018	GB	CCRT + adj TMZ (×1)	1	STR + ICI (×7) + FSRT	Stable	-
6	F/48	STR/October 2018	GB	CCRT	12	STR + ICI (×18) + FSRT	Progression	2
7	M/47	STR/November 2018	GB	CCRT + adj TMZ (×1)	1	ICI (×8) + FSRT	Progression	2
8	F/54	STR/January 2019	GS	CCRT + adj TMZ (×3)	1	STR + ICI (×6) + FSRT	Stable	-

Progression was detected on brain MRI in all patients with a median of 5.8 months (range: 1-17.4 months) after EBRT. Patients 1, 5, 6, and 8 underwent STR and the final pathologies were the same as the first ones. All patients received three cycles of nivolumab prior to FSRT and underwent 3 × 8 Gy FSRT with concurrent and adjuvant nivolumab with a median of 9.5 months (range: 3-25 months) after EBRT. The Median GTV volume at FSRT was 37 cm^3^ (range: 18-123 cm^3^), and the prescribed isodose was a median of 86.5% (range: 81-88%). The median number of total nivolumab cycles was 8 (range: 6-18 cycles).

**Figure 1:**
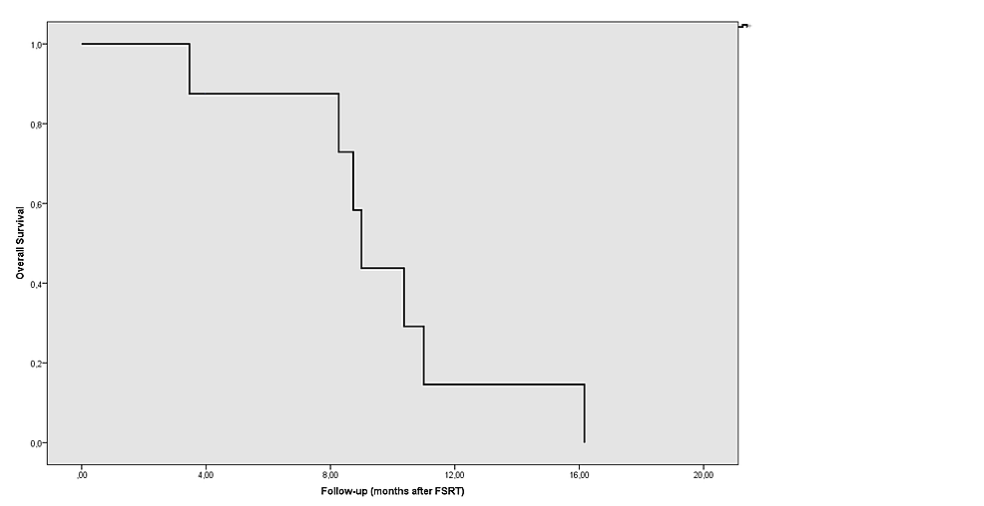
Overall survival after FSRT. FSRT: fractionated stereotactic radiotherapy

**Figure 2:**
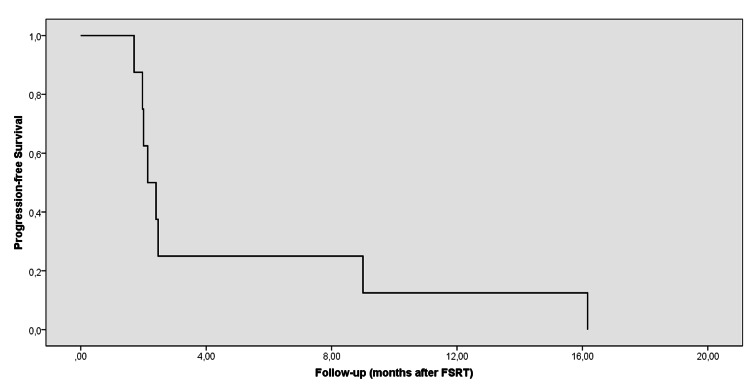
Progression-free survival after FSRT FSRT: fractionated stereotactic radiotherapy

Median follow-up was 21.3 months (range: 8.6-35.3 months) from the first diagnosis and 12.6 months (range: 6.1-18.5 months) from recurrence. Seven patients succumbed to the disease. Only patient number 6 is alive after a follow-up of 21.6 months after the first diagnosis and 7.8 months after progression. The rate of one- and two-year OS was 88% and 33%, respectively, and median OS was 20.9 months (95% confidence interval [CI] = 17.8-24.1; standard error [SE] = 1.6) after the first diagnosis. Following progression, the one- and two-year OS rate was 73% and 0%, respectively (Figure 1), and median OS was 12.9 months (95% CI = 11.4-14.4; SE = 0.8). The Median progression-free interval was 2.3 months (range: 1.7-16.2 months) after FSRT. In three patients who recurred in less than three months after the first EBRT, the median OS after recurrence was 12.5 months (range: 6.1-18.5 months). LC was achieved in five patients after FSRT, with the LC rate being 62.5%. The one-year LRFS rate was 14.6%, and the median LRFS was nine months (95% CI = 8.3-9.7; SE = 0.34) after FSRT. However, despite LC, the disease recurred in other regions of the brain in patients 2, 3, 4, and 8. Patient number 8 also developed leptomeningeal and spinal spread three months after FSRT and received 36 Gy EBRT to the whole spinal cord and 45 Gy to the gross tumors in the spinal cord. The overall one-year PFS rate was 12.5% (Figure 2), and the median PFS was 2.1 months (95% CI = 1.6-2.7; SE = 0.3) after FSRT.

The median follow-up after the first dose of nivolumab was 10.3 months (range: 5.2-17.9 months). Following one cycle, three (37.5%) patients (3, 5, and 7) demonstrated PR, two (25%) (4 and 6) demonstrated stable disease, and three (37.5%) (1, 2, and 8) demonstrated progressive disease. The one- and two-year rate of PFS after the start of nivolumab was 12.5% and 0%, respectively, and the median PFS was 3.9 months (95% CI = 1.22-6.6; SE = 1.36). The rate of OS after progression did not differ among patients with PR, stable disease, and progression.

Acute toxicity was not observed during FSRT. Grade 3 pneumonitis was observed in patients 5 and 7 who received seven and eight cycles of nivolumab, respectively. Patient number 5 who was re-irradiated five months after EBRT also experienced severe osteoporosis and radiation necrosis. In these two patients, nivolumab had to be discontinued. Grade 1 thyroid toxicity developed in patients 4 and 8 who received six cycles of nivolumab (Table 1). There were no treatment-related deaths.

## Discussion

This is the second study to report the outcomes of combined ICI and FSRT in recurrent high-grade gliomas. However, the FSRT and ICI regimens differ in these two studies [10]. An LC rate of 62.5% was achieved in our study. Although most failures were in the EBRT field during the first recurrence, most failures were in the FSRT field in the following recurrence. Combined ICI and FSRT could be applied in most patients without severe toxicity. In 2014, we published our results in 37 patients with recurrent GB treated with a median 30 Gy FSRT in five fractions [8]. The median survival was 10.6 months after FSRT which is lower than that of the current study. The most striking finding in the current study is the dominance of out-of-FSRT-field recurrences in the next failure; however, it was mostly in the FSRT field in our previous study. It is important to note that not only the OS increases with FSRT+ICI but the failure pattern is also modified.

The literature on ICI in brain tumors has gained attention recently. Chamberlain et al. reported the outcomes of 16 patients with GB who received nivolumab after progression on bevacizumab [15]. Of the 16 patients, seven progressed after the first cycle in whom the treatment was stopped, and stable disease was assessed in the remaining. The median OS and PFS after progression were 3.5 months and 2 months, respectively. The median OS from diagnosis was 16.25 months, and nivolumab had no survival advantage. In the CheckMate 143 trial, 369 patients with a first recurrence of GB were randomized to nivolumab or bevacizumab [16]. The median OS was 9.8 months and 10 months, median PFS was 1.5 months and 3.5 months, and the rates of CR and PR were 1% and 6.5% after nivolumab and bevacizumab, respectively. Both studies used ICI alone. In our study, a longer median OS of 12 months was found with FSRT+ICI. Even in patients who recurred shortly after the first EBRT, the median OS was 12.5 months.

The combination of RT and PD-1 inhibitors significantly prolongs survival compared to either treatment alone [17]. Both preclinical and clinical trials have shown that RT upregulates the expression of PD-L1 [18,19]. Radiation leads to immune activation via many pathways and reduces the time to response and improves the responsiveness of tumors refractory to anti-PD1/PD-L1 monotherapy [20,21]. It also increases the permeability of the blood-brain barrier and the penetrance of systemic agents and activated antitumor immune cells into the CNS [22]. However, modern techniques such as IMRT and VMAT apply low doses to larger volumes which can decrease the immune response by affecting circulating lymphocytes [23]. The timing of RT, irradiated volume, and fractionation scheme can also affect the immune response. Hypofractionated ablative RT decreases the recruitment of myeloid-derived suppressor cells which exhibit immunosuppressive properties leading to tumor progression and the establishment of a premetastatic niche [24]. Grapin et al. irradiated mice with brain tumors with 18 × 2 Gy, 3 × 8 Gy, and 1 × 16.4 Gy which have equal biologically effective dose (BED) values, and injected anti-PD-L1 and anti-T-cell immunoreceptor with immunoglobulin and immunoreceptor tyrosine-based inhibitory motif domains (anti-TIGIT) three times a week, starting on the first day of RT [25]. A higher tumor growth delay was observed with fractionated schemes compared to a single fraction. With the addition of anti-PD-1 alone, 3 × 8 Gy was the most effective treatment. Preclinical data have suggested that hypofractionated RT of 5-20 Gy per fraction may be better than conventional fractionated schemes [26]. Based on these findings, we administered 3 × 8 Gy FSRT with ICI to induce a stronger immune response.

A recent phase I study was completed in which bevacizumab and ICI were initiated at the same time with FSRT and continued for five cycles [10]. The main differences of this study from ours were the 5 × 6 Gy FSRT regimen, pembrolizumab used with no neoadjuvant prior to FSRT, and the addition of bevacizumab based on which patients were grouped as naïve and resistant and compared. Predominantly based on the RANO criteria, the authors reported a median OS of 13.45 months and 9.3 months in bevacizumab-naïve and -resistant patients, respectively, which is similar to our findings. The one- and two-year OS rate was 91.7% and 58% and 88% and 25% in naïve and resistant patients, respectively. Here, we report a one-year OS rate of 73% in all patients. The control rate in that study was 100% and 75% in the respective group of patients after a median of 3.7 months and 3.1 months of follow-up, which is significantly lower than ours of 10.3 months. Local progression was observed in two patients only, who were both bevacizumab-resistant. However, it is unclear why patients succumbed and where the disease recurred. The PFS rates were only given for naïve patients which were 29% in one year. We report a one-year PFS rate of 12.5% which would probably be similar to the results of the previous study if the rate for resistant patients was provided.

The most common side effects of ICI are related to the inflammatory and autoimmune response in the skin, colon, liver, and lungs, which are more common with ipilimumab, and discontinuation of treatment was more often than with nivolumab (13.2% vs. 5.1%) [27]. For the CNS tumors, the potential role of ICI on inflammatory and autoimmune events, such as allergic encephalomyelitis, increased intracranial pressure, and cerebral edema, which can reduce tolerability, should be approached with caution [28]. When combined with RT, toxicity rates may increase due to the synergistic stimulation of both local and systemic immunities. We did not observe any intracranial immune-related toxicity. However, grade 3 pneumonitis was observed in two and mild thyroid toxicity in another two patients. Chamberlain et al. observed grade 3 adverse events in two patients after a median of two cycles of nivolumab [15]. Following a median of three cycles of nivolumab in the CheckMate 143 trial, the rate of grade 3-4 adverse events was 18%, which was not significantly different from that of bevacizumab [16]. In our study, grade 3 nivolumab-related pneumonitis was observed in two (25%) patients. Although the rate appears to be higher, it should be noted that the median number of nivolumab cycles was higher in our study. In the study of Sahebjam et al., the rate of grade 3 toxicity was 34% [10]. The authors reported no radiation necrosis but mild thyroid function disorders in 25% and pneumonitis-related symptoms in 6% of the patients. We also observed mild thyroid disorders in two (25%) patients. The higher rate of pneumonitis in our study may be due to the concurrent use of bevacizumab as in the study of Sahebjam et al., which may have belied some of the adverse events [10]. Further, median GTV volume (37 cm^3^ vs. 9.9 cm^3^) and the number of ICI cycles (eight vs. five) were higher in our study.

There are still many unknown factors with the use of immunoradiotherapy such as the optimal patient selection, optimal ICI, optimal sequencing of ICI and RT, and the toxicity of ICI alone or in combination with RT. When the potential synergism is taken into account, initiation of ICI prior to RT seems rational. However, the majority of clinical trials have utilized concurrent RT and ICI. In a preclinical study by Dovedi et al., mice with brain tumors were irradiated with 5 × 2 Gy with anti-PD-L1 given on the first or the last day of RT or seven days after the last irradiation [29]. Concurrent use significantly increased the OS rate whereas it was not affected by sequential treatment. In the PACIFIC trial on nonsmall cell lung cancer, patients who received ICI in <14 days closer to RT benefited more compared to >14 days [30]. Based on these data, we administered neoadjuvant and concurrent ICI with FSRT and continued ICI following FSRT.

The limitations of the current case series include the varying number of nivolumab cycles, lack of knowledge regarding PD-L1 level, and the timing of steroid use which may interfere with immunotherapy efficacy. Besides, as we could not analyze the MGMT and mismatch repair status in all patients, we could not compare the efficacy of our treatment in regard to these parameters. However, we achieved an LC rate of 62.5% in recurrent high-grade gliomas after 3 × 8 Gy FSRT+ICI. Severe toxicity was observed as radiation necrosis in one patient and pneumonitis in two patients.

## Conclusions

Although the concurrent use of FSRT and ICI does not yield significant results in recurrent high-grade gliomas, it can be an option. This combined treatment is successful in controlling the local disease but not for other failures in the brain. Neoadjuvant use of ICI prior to FSRT may have also been responsible for the good LC rate. On the other hand, ICI-related toxicity cannot yet be foreseen. Numerous clinical trials on the role of various ICIs on newly diagnosed and recurrent GB and other brain tumors are ongoing and the results are awaited with great interest.

## References

[1] Stupp R, Mason WP, van den Bent MJ (2005). Radiotherapy plus concomitant and adjuvant temozolomide for glioblastoma. N Engl J Med.

[2] Van den Bent MJ, Erridge S, Vogelbaum MA (2016). Results of the interim analysis of the EORTC randomized phase III CATNON trial on concurrent and adjuvant temozolomide in anaplastic glioma without 1p/19q co-deletion: an intergroup trial. J Clin Oncol.

[3] Gilbert MR, Dignam JJ, Armstrong TS (2014). A randomized trial of bevacizumab for newly diagnosed glioblastoma. N Engl J Med.

[4] Chinot OL, Wick W, Mason W (2014). Bevacizumab plus radiotherapy-temozolomide for newly diagnosed glioblastoma. N Engl J Med.

[5] Neville IS, Dos Santos AG, Almeida CC (2021). Reoperation for recurrent glioblastomas: what to expect?. Surg Neurol Int.

[6] Fogh SE, Andrews DW, Glass J (2010). Hypofractionated stereotactic radiation therapy: an effective therapy for recurrent high-grade gliomas. J Clin Oncol.

[7] Combs SE, Thilmann C, Edler L, Debus J, Schulz-Ertner D (2005). Efficacy of fractionated stereotactic reirradiation in recurrent gliomas: long-term results in 172 patients treated in a single institution. J Clin Oncol.

[8] Yazici G, Cengiz M, Ozyigit G (2014). Hypofractionated stereotactic reirradiation for recurrent glioblastoma. J Neurooncol.

[9] Belcaid Z, Phallen JA, Zeng J (2014). Focal radiation therapy combined with 4-1BB activation and CTLA-4 blockade yields long-term survival and a protective antigen-specific memory response in a murine glioma model. PLoS One.

[10] Sahebjam S, Forsyth PA, Tran ND (2021). Hypofractionated stereotactic re-irradiation with pembrolizumab and bevacizumab in patients with recurrent high-grade gliomas: results from a phase I study. Neuro Oncol.

[11] Mallick S, Benson R, Hakim A, Rath GK (2016). Management of glioblastoma after recurrence: a changing paradigm. J Egypt Natl Canc Inst.

[12] Wen PY, Macdonald DR, Reardon DA (2010). Updated response assessment criteria for high-grade gliomas: response assessment in neuro-oncology working group. J Clin Oncol.

[13] Okada H, Weller M, Huang R (2015). Immunotherapy response assessment in neuro-oncology: a report of the RANO working group. Lancet Oncol.

[14] Louis DN, Perry A, Reifenberger G (2016). The 2016 World Health Organization classification of tumors of the central nervous system: a summary. Acta Neuropathol.

[15] Chamberlain MC, Kim BT (2017). Nivolumab for patients with recurrent glioblastoma progressing on bevacizumab: a retrospective case series. J Neurooncol.

[16] Reardon DA, Brandes AA, Omuro A (2020). Effect of nivolumab vs bevacizumab in patients with recurrent glioblastoma: the CheckMate 143 phase 3 randomized clinical trial. JAMA Oncol.

[17] Zeng J, See AP, Phallen J (2013). Anti-PD-1 blockade and stereotactic radiation produce long-term survival in mice with intracranial gliomas. Int J Radiat Oncol Biol Phys.

[18] Deng L, Liang H, Burnette B, Weicheslbaum RR, Fu YX (2014). Radiation and anti-PD-L1 antibody combinatorial therapy induces T cell-mediated depletion of myeloid-derived suppressor cells and tumor regression. Oncoimmunology.

[19] Twyman-Saint Victor C, Rech AJ, Maity A (2015). Radiation and dual checkpoint blockade activate non-redundant immune mechanisms in cancer. Nature.

[20] Lugade AA, Sorensen EW, Gerber SA, Moran JP, Frelinger JG, Lord EM (2008). Radiation-induced IFN-gamma production within the tumor microenvironment influences antitumor immunity. J Immunol.

[21] Reits EA, Hodge JW, Herberts CA (2006). Radiation modulates the peptide repertoire, enhances MHC class I expression, and induces successful antitumor immunotherapy. J Exp Med.

[22] van Vulpen M, Kal HB, Taphoorn MJ, El-Sharouni SY (2002). Changes in blood-brain barrier permeability induced by radiotherapy: implications for timing of chemotherapy? (Review). Oncol Rep.

[23] Tang C, Liao Z, Gomez D (2014). Lymphopenia association with gross tumor volume and lung V5 and its effects on non-small cell lung cancer patient outcomes. Int J Radiat Oncol Biol Phys.

[24] Lan J, Li R, Yin LM (2018). Targeting myeloid-derived suppressor cells and programmed death ligand 1 confers therapeutic advantage of ablative hypofractionated radiation therapy compared with conventional fractionated radiation therapy. Int J Radiat Oncol Biol Phys.

[25] Grapin M, Richard C, Limagne E (2019). Optimized fractionated radiotherapy with anti-PD-L1 and anti-TIGIT: a promising new combination. J Immunother Cancer.

[26] Sharabi AB, Lim M, DeWeese TL, Drake CG (2015). Radiation and checkpoint blockade immunotherapy: radiosensitisation and potential mechanisms of synergy. Lancet Oncol.

[27] Michot JM, Bigenwald C, Champiat S (2016). Immune-related adverse events with immune checkpoint blockade: a comprehensive review. Eur J Cancer.

[28] Heimberger AB, Sampson JH (2011). Immunotherapy coming of age: what will it take to make it standard of care for glioblastoma?. Neuro Oncol.

[29] Dovedi SJ, Adlard AL, Lipowska-Bhalla G (2014). Acquired resistance to fractionated radiotherapy can be overcome by concurrent PD-L1 blockade. Cancer Res.

[30] Antonia SJ, Villegas A, Daniel D (2017). Durvalumab after chemoradiotherapy in stage III non-small-cell lung cancer. N Engl J Med.

